# Does early adversity predict aggression among Chinese male violent juvenile offenders? The mediating role of life history strategy and the moderating role of meaning in life

**DOI:** 10.1186/s40359-023-01407-9

**Published:** 2023-11-08

**Authors:** Yuandong Gong, Qingtian Li, Jiazheng Li, Xinning Wang, Wenning Jiang, Weiguo Zhao

**Affiliations:** 1 Shandong Mental Health Center, Shandong University, Jinan, China; 2 School of Psychology, Shandong Normal University, No. 1, Daxue Road, Changqing District, Jinan, Shandong China; 3 School of Psychology and Educational Sciences, Zaozhuang University, No. 1, Beian Road, Shizhong District, Zaozhuang, Shandong China; 4 Shanghai Shuguang High School of Fengxian District, No. 100, Chuanbo Road, Fengcheng Town, Fengxian District, Shanghai, China

**Keywords:** Aggression, Early adversity, Life history strategy, Meaning in life, Chinese male violent juvenile offenders, Moderated mediation model

## Abstract

**Background:**

Adolescent aggression has long been of interest to researchers. However, few studies have examined the influencing factors and mechanisms of aggression among violent juvenile offenders. This study tests a moderated mediation model with Chinese male violent juvenile offenders as subjects. Specifically, it explores the relationship between early adversity and aggression, as well as the mechanisms of life history strategy and meaning in life in this relationship.

**Methods:**

A total of 537 Chinese male violent juvenile offenders completed the Childhood Environment Scale, the Life History Strategy Short Form Scale, the Aggression Questionnaire, and the Meaning in Life Questionnaire. After controlling for socioeconomic status (SES), the current cross-sectional study used structural equation modeling (SEM) to examine a moderated mediation model.

**Results:**

The results showed that life history strategy mediated the relationship between early adversity and aggression, and early adversity affected individuals’ aggression by accelerating their life history strategies. The results also showed that meaning in life moderated the relationship between early adversity and life history strategy. For individuals with high meaning in life scores, the negative predictive effect of early adversity on life history strategy was stronger than that for individuals with low meaning in life scores.

**Conclusion:**

The results of this study can advance the understanding of how early adversity affects aggression among violent juvenile offenders and provide theoretical support for prison staff to develop educational strategies and subsequent interventions.

## Introduction

Aggression in the adolescent population(14–25 years old) has been an important topic of concern in psychology. According to the “White Paper on Juvenile Prosecutorial Work (2021)” issued by the Supreme People’s Procuratorate of the People’s Republic of China [1], violent juvenile offenders are the main juvenile offenders (accounting for more than 40% of the total). Violent juvenile offenders are more likely to engage in aggression than other groups [2, 3]. Aggression not only predicts the risk of recidivism but also seriously endangers others and their own physical and mental health, which then impacts their rehabilitation in prison and the stability of social security [4, 5]. Therefore, it is essential to explore the influencing factors and psychological mechanisms of violent juvenile offenders’ aggression to help prevent juvenile delinquency and reduce the risk of recidivism among violent juvenile offenders, as well as the risk of them committing homicide or suicide while serving their sentences.

Problem behavior theory suggests that the occurrence of problem behavior involves three systems: the perceived environmental system, the personal system, and the behavioral system [6]. This theory provides a fundamental theoretical framework paradigm for the occurrence of aggression. Individual behavior will be affected by the environment system and personal system during evolution [7]. Among these, early adversity is considered a constant risk factor that shapes an individual’s life history strategy and triggers individual aggression [8]. Regarding personal systems, the view of positive youth development emphasizes the effect of positive psychological qualities on individual developmental outcomes. Among them, meaning in life is considered an important protective factor for individuals in the face of adverse circumstances [9].

In real-life situations, aggression is often the result of multiple systems or factors acting simultaneously. In contrast, previous studies exploring the mechanisms of aggression have focused on the role of a single system (e.g., environmental system or personal system) or factor (e.g., dangerous factor or protective factor) in aggression while ignoring the simultaneous effects among multiple systems or factors. In addition, although the relationship between early adversity and aggression has been well studied, existing studies have mainly focused on typical adolescents but rarely involved violent juvenile offenders [10]. Thus, this study tests the simultaneous impact of early adversity, life history strategy, and meaning in life on violent juvenile offender aggression to understand the relationship between these antecedent and consequence variables and their mechanisms of action.

### The relationship between early adversity and aggression

The negative effects of early adversity on adolescents have been widely noted. Empirical research suggests that approximately one- to two-thirds of juvenile offenders experience some form of early adversity [11]. Those who experience early adversity are more aggressive than those with no experience with adversity [12]. In addition, adverse childhood experiences are associated with certain antisocial personality traits that predict aggression [10, 13]. Studies from several Asian countries, including China, India, and Japan, have also demonstrated that the adversarial childhood maltreatment of prison inmates significantly and positively predicts aggression in violent juvenile offenders [14–16]. In summary, this study proposes hypothesis 1: Early adversity significantly and positively predicts violent juvenile offenders’ aggression.

### Life history strategy as a mediator

Throughout the entire process of life, humans inevitably face limited resources, such as money, time, and so on. Life history strategies reflect an individual’s tendency to balance resource allocation when facing limited resources in the environment. They are a collection of individual specific physiological, psychological, and behavioral patterns that belong to the internal system elements of the individual [17, 18]. This mode mainly includes two strategies: fast and slow on a continuum. Examples of fast strategies include precocious puberty, early reproductive age, pursuit of short-term goals, and reluctance to delay gratification. Examples of slow strategies include late maturity, delayed reproductive age, pursuit of long-term goals, and a greater willingness to delay gratification [7]. Balancing and adaptation are two major themes in life history, which correspond to the causes and consequences of individual life history strategy choices and are a coherent process. The so-called trade-off refers to the allocation of resources by individuals when facing them, that is, the process of selecting life history strategies in their life process. Adaptation refers to the developmental outcomes exhibited by individuals after choosing life history strategies [19]. From this, it can be seen that the life history strategy is the core of life history theory, undertaking the balance between early life and later development results.

According to life history theory, from an evolutionary perspective, in order to achieve maximum development, individuals need to make different trade-offs in front of limited resources, including the four major trade-offs of “Trade off between maintenance and growth”, “Present-future production trade off”, " Trade-off between mating effort and parenting effort “, and “Quantity quality of offspring trade off”. These trade-off issues are to some extent determined by the environmental characteristics of an individual’s early life. When an individual’s early growth environment is safe and predictable, they focus their energy and resources on developmental tasks and have more long-term strategies, such as learning knowledge and investing more in the upbringing of future generations. The harsh and unpredictable childhood environment may lead to individuals focusing their energy and resources on survival tasks and having more short-term strategies, such as precocious puberty Investing less in offspring rearing and adopting a faster life history strategy [17, 20, 21]. Empirical studies also confirm that the early life environment of individuals (such as lower childhood economic and social status) leads individuals to form a fast life strategy [22]. Conversely, deterministic, secure, and stable environments facilitate the formation of a slow life history strategy [23].

Individuals are more likely to form fast life history strategies if they have limited resources and live in harsh and unpredictable environments. This life history strategy is implemented through behaviors with the following characteristics: greater randomness, risk-taking [24], and impulsivity and less adherence to social and moral norms and laws [25, 26]. Among violent juvenile offenders, these behavioral characteristics may manifest as a fast life history strategy guided by committing more aggression to obtain more significant gains. In a recent longitudinal study of Chinese left-behind children, researchers found that the unpredictable environmental factor of parental separation was more likely to lead individuals to develop fast life history strategies, and thus exhibit more aggressive and risk-taking behaviors, than to develop slow life history strategies [27].

In summary, this study proposes hypothesis 2: Life history strategy mediates the relationship between early adversity and aggression among violent juvenile offenders.

### Meaning in life as a moderator

The positive development perspective emphasizes not only paying attention to pathological issues, but also paying attention to individual strengths in growth [28]. The group of violent juvenile offenders has earlier exposure to society and may have a more harsh living environment, with less interaction with social culture [29–31]. This characteristic determines that the group’s understanding and pursuit of the meaning of life is one of its basic needs [32]. Exploring the significance of life is particularly important for the development of this group. Meaning in life is the individual’s ability to comprehend and understand life’s meaning and explore and pursue goals. It is an essential motivating force for self-actualization and a critical element of the personal system [33].

According to personal-context interaction theory, the relationship between environmental risk factors(early adversity) and developmental outcomes(life history strategy、aggression) may be moderated by positive personal traits(meaning in life) [34]. Protective factors interact with risk factors in two typical moderation models. First, in the “enhancement model”, protective factors weaken the adverse effects of risk factors [35]. For example, Lu and Gong found that meaning in life buffered the impact of loneliness on smartphone addiction among a group of 408 Chinese university students [36]. Second, the “attenuation model” proposes that protective factors are not sufficient to cushion the negative impact of risk factors, and risk factors weaken their protective effect, the protective effect of protective factors will be stronger than when the risk factors are higher [37]. For example, researchers have found that for individuals with high meaning in life, the negative predictive effect of emotional neglect on problematic network use is more significant than that for individuals with low meaning in life [38].

As mentioned above, there is inconsistency in the results. Moreover, most of these studies have used the general population as subjects. The moderation mechanism between early adversity and developmental outcomes for meaning in life for violent juvenile offenders is unclear (consistent with an enhancement or attenuation model). Thus, this study does not make specific inferences about the moderating direction of meaning in life but proposes hypothesis 3: Meaning in life moderates the relationship between early adversity and life history strategy and moderates the relationship between life history strategy and aggression among violent juvenile offenders.

### Current study

**Fig. 1:**
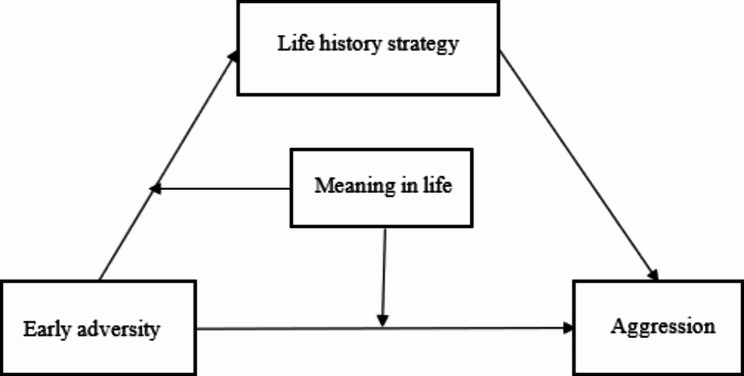
A moderated mediation model

This study uses the three systems of problem behavior theory as the basic framework. From the perspective of life history theory and the view of positive youth development, a moderated mediation model (Fig. 1) is proposed to uncover the risk and protective factors of aggression among Chinese male juvenile offenders. This study attempts to provide an empirical basis for the subsequent development of intervention strategies for aggression among violent juvenile offenders.

## Methods

### Participants and procedure

The sample included 537 male juvenile offenders from detention centers in Sichuan Province, China. In addition to sexual and drug offenses, the main types of crimes included manslaughter, assault, and battery. This study ended up with 491 valid questionnaires. In terms of demographic variables, this study investigated the family socioeconomic status, age, and gender of participants. The age range of the participants was 14–25 years old (*M* = 19.29, *SD* = 2.40), and all of them were male. Data on parents’ gross monthly income and education level were used to calculate the SES index.

Approval was obtained from the Ethics Committee of Shandong Normal University. All procedures performed in the study were in accordance with the 1964 Helsinki declaration and its later amendments or comparable ethical standards. Informed consent was obtained from the Administrator of Juvenile Detention Centers, the participants’ parents and the participants themselves. The data were collected by means of a questionnaire booklet. The questionnaire was completed in 15–30 min, and anonymity and confidentiality were ensured. Additionally, this process was completed under the supervision of the psychological research assistants.

### Measures

#### Early adversity

Early adversity was measured using 14 items(e.g. How many times has your parent or caregiver failed to give you basic needs, such as food) from the Childhood Environment Scale, which was developed by Ellis et al. and revised by Chen and Chang [21, 39]. The questionnaire was divided into two dimensions: harshness and unpredictability. Participants were asked to recall their childhood experiences before the age of 12. The questionnaire is scored on four points, ranging from 1 (never) to 4 (always), and the Cronbach’s α = 0.85.

#### Life history strategy

The measurement of the life history strategy involved the Life History Strategy Short Form Scale (Mini-k), which was developed by Figueredo et al. and was revised by Chen et al [40, 41]. The scale was developed by has 20 items(e.g. I never give up until the problem is solved). Given that this study is examining the characteristics of juvenile delinquents, the items related to sexual partners are excluded from the scale (for example, “I prefer fixed sexual partners to maintaining sexual relations with multiple people”). Ultimately, 15 items are retained. Participants were invited to use a seven-point scoring scale to express their life history strategy speed, from 1 (totally disagree) to 7 (totally agree). The total score of all questions represents the score of the life history strategy. The higher the score, the slower the life history strategy. Cronbach’s α = 0.84. CFA(Confirmatory Factor Analysis) results show that the model fits the data well, *χ*^2^/*df* = 3.13, RMSEA = 0.07, GFI = 0.94, NFI = 0.91, TLI = 0.91, CFI = 0.93.

#### Aggression

Aggression was measured using 34 items(e.g. Sometimes I get angry and angry for no reason) from the Aggression Questionnaire, which was revised by Maxwell in Chinese sample [42]. The questionnaire was divided into five dimensions: physical aggression, verbal aggression, indirect aggression, anger, and hostility. And it is scored by five points, from 1 (strongly opposed) to 5 (strongly agreed). Cronbach’s α = 0.93.

#### Meaning in life

Meaning in life was measured using 9 items(e.g. My life has a clear direction) from the Meaning in Life Questionnaire, which was prepared by Steger et al [43], and revised by Liu and Gan [44]. The questionnaire was divided into two dimensions: the presence of meaning and the search for meaning in life. The questionnaire is scored on seven points, ranging from 1 (absolutely untrue) to 7 (absolutely true), and the Cronbach’s α = 0.88.

### Statistical analyses

AMOS 24.0 was used for the validation factor analysis of the questionnaire. Other analyses were performed using IBM SPSS Statistics version 26. Missing data (less than 1%) were first processed using the expectation-maximization algorithm.

## Results

### Preliminary analyses

The results of the correlation analysis (Table 1) showed that SES was significantly and positively correlated with life history strategy, aggression, and meaning in life. Early adversity was significantly and negatively correlated with life history strategy and meaning in life and significantly and positively correlated with aggression. Aggression was significantly and negatively correlated with life history strategy; meaning in life was significantly and positively correlated with life history strategy. Age was irrelevant to all variables. SES is used as the control variable for subsequent analyses.

**Table 1:** Descriptive statistics and correlation analysis of the variables (*n* = 491)

	1	2	3	4	5	6
1. SES						
2. Age	-0.03					
3. Early adversity	0.07	0.08				
4. Life history strategy	0.13^*^	-0.06	–0.21^**^			
5. Aggression	0.13^**^	-0.01	0.44^**^	–0.22^**^		
6. Meaning in life	0.10^*^	0.02	–0.12^**^	0.37^**^	–0.06	
*M*	0.00	19.28	1.61	63.20	2.36	4.93
*SD*	0.81	2.40	0.41	13.47	0.62	1.26

Note: ^*^p < 0.05, ^**^p < 0.01, ^***^p < 0.001

### Testing for the mediation model

First, all variables were standardized, and then the mediating effects were tested while controlling SES. The results showed (Table 2) that early adversity significantly and positively predicted aggression (*β* = 0.43, *t* = 10.66, *p* < 0.001), the total effect was significant, and hypothesis 1 holds. The direct effect of early adversity on aggression remained significant (*β* = 0.40, *t*= − 5.04, *p* < 0.001); meanwhile, early adversity significantly and negatively predicted life history strategy (*β*= − 0.22, *t* = 9.73, *p* < 0.001), and life history strategy significantly and negatively predicted aggression (*β*= − 0.15, *t*= − 5.36, *p* < 0.01). The indirect effect ab = 0.03, accounting for 7% of the total effect, *Boot SE* = 0.01, with a 95% CI[0.01,0.06], suggests that life history strategy partially mediates the relationship between early adversity and aggression. Research hypothesis 2 holds.

**Table 2:** Tests of mediating effect (*n* = 491)

Predictors	Equation 1: Life history strategy(M)					Equation 2: Aggression(Y)			
	*β*	*SE*	*t*	*95% CI*		*β*	*SE*	*t*	*95% CI*
SES	0.15	0.04	3.37^**^	[0.06,0.23]		0.12	0.04	2.86^**^	[0.04,0.20]
Early adversity(X)	–0.22	0.04	–5.04^***^	[–0.31, − 0.13]		0.40	0.04	9.73^***^	[0.32,0.48]
Life history strategy				[0.26, 0.42]		–0.15	0.04	–5.36^**^	[–0.23, − 0.06]
*R* ^*2*^	0.07					0.22			
*F*	17.23^***^					46.31^***^			

Note: ^*^p < 0.05, ^**^p < 0.01, ^***^p < 0.001

### Testing for the moderated mediation model

The moderated mediator model was tested using Model 8 of the SPSS macro program. The results showed (Table 3) that in Eq. 1, early adversity significantly and negatively predicted life history strategy (*β*= − 0.20, *t*= − 4.68, *p* < 0.001), and the interaction term between early adversity and meaning in life in the predictive effect of life history strategy holds (*β*= − 0.09, *t*= − 2.43, *p* < 0.05), which indicates that meaning in life plays a moderating role in the impact of early adversity on life history strategy. Equation 2 showed that early adversity significantly and positively predicted aggression (*β* = 0.41, *t*= − 9.76, *p* < 0.001); life history strategy significantly and negatively predicted aggression (*β*= − 0.16, *t*= − 3.54, *p* < 0.01); and the interaction term between early adversity and meaning in life was not significant in predicting aggression (*β*= − 0.04, *t* = 1.03, *p* > 0.05), indicating that meaning in life does not play a moderating role in the impact of early adversity on aggression. In summary, meaning in life plays a moderating role in the first half of the mediating effect.

**Table 3:** The test of the moderated mediator (*n* = 491)

Predictors	Equation 1: Life history strategy(M)					Equation 2: Aggression(Y)			
	*β*	*SE*	*t*	*95% CI*		*β*	*SE*	*t*	*95% CI*
SES	0.11	0.04	2.64^**^	[0.03,0.19]		0.11	0.04	2.81^**^	[0.03,0.19]
Early adversity(X)	–0.20	0.04	–4.68^***^	[–0.28, − 0.11]		0.41	0.04	9.76^***^	[0.33,0.49]
Meaning in life(W)	0.34	0.04	8.16^***^	[0.26, 0.42]		0.04	0.04	0.93	[–0.04,0.13]
X×W	–0.09	0.04	–2.43^*^	[–0.17, − 0.02]		0.04	0.04	1.03	[–0.04, 0.11]
Life history strategy						–0.16	0.04	–3.54^**^	[–0.24, − 0.07]
*R* ^*2*^	0.19					0.23			
*F*	27.96^***^					28.19^***^			

Note: ^*^p < 0.05, ^**^p < 0.01, ^***^p < 0.001

Further overall model testing of the moderated mediating effects (Table 4) revealed that the mediating effect of life history strategy between early adversity and aggression was significant when meaning in life scores was one standard deviation below the mean (*Effect* = 0.02, *95% CI*=[0.01, 0.04]). The mediating effect of life history strategy was significant. It was enhanced when meaning in life scores were one standard deviation above the mean (*Effect* = 0.05, *95% CI*=[0.02,0.09]), again validating the mediating model with moderation.

**Table 4:** Bootstrap test of moderated mediator

Moderator	Level	effect	Boot *SE*	Low Bootstrap	Up Bootstrap
Meaning in life	*M –* 1 *SD*	0.02	0.02	0.01	0.04
	*M*	0.03	0.03	0.01	0.06
	*M +* 1 *SD*	0.05	0.02	0.02	0.09

### Simple slope analysis

**Fig. 2:**
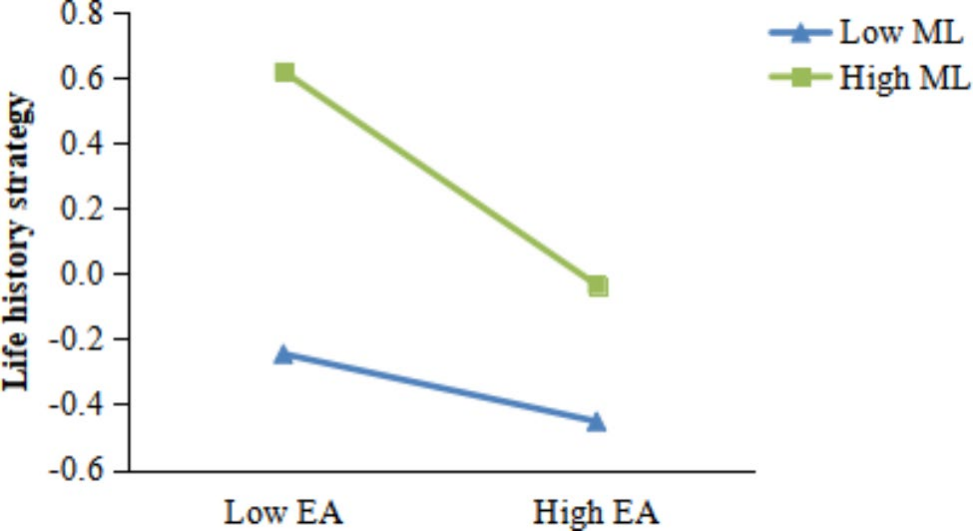
The moderating effect of meaning in life between early adversity and life history strategy Note: EA = early adversity; ML = meaning in life.

Respondents were divided according to meaning in life into a high group (*M + 1SD*) and a low group (*M-1SD*), and then a simple slope analysis was conducted for both groups. The results showed that (Fig. 2). When the level of meaning in life is low, as the level of early adversity decreases, the negative predictive effect of early adversity on life history strategies is significant(*β*_simple (M−1SD)_ = − 0.10, *t* = − 2.01, *p* < 0.05, *95% CI* = [ − 0.20, − 0.01]); When the level of meaning in life is high, as the level of early adversity decreases, the negative predictive effect of early adversity on life history strategies is greater(*β*_simple (M−1SD)_= − 0.30, *t* = − 4.66, *p* < 0.001), *95% CI*=[–0.41, − 0.17]). As the level of early adversity decreases, individuals with a high meaning in life are more likely to adopt a slow life history strategy compared to individuals with a low meaning in life. This indicates that the protective effect of the meaning in life is stronger at low early adversity, supporting the attenuation model.

## Discussion

This study explored the relationship between early adversity and aggression, the mediating role of life history strategy, and the moderating role of meaning in life, further revealing the mechanisms and moderators of aggression in violent juvenile offenders. The protective effect of meaning in life was limited, and meaning in life was able to buffer the relationship between early adversity and life history strategy only at low early adversity levels.

### The relationship between early adversity and aggression

Early adversity significantly and positively predicted aggression among violent juvenile offenders. This result is consistent with previous studies [14]. A possible explanation for this result can be adopted from social learning theory. Adolescents acquire aggression in hostile environments, such as chronic exposure to domestic violence and emotional life. They rationalize and interpret this behavior and internalize it as one of their main ways of interacting with others [45]. Another possible explanation is that early adversity contributes to the formation of antisocial personality traits such as impulsivity and angry ruthlessness that make individuals more aggressive during adolescence [2, 46].

### The mediating role of life history strategy

This study found that life history strategy partially mediates the relationship between early adversity and aggression among violent juvenile offenders. This finding is consistent with the study of Lu and Chang [27]. From a biological evolutionary perspective, the harshness and unpredictability of the environment increase the uncertainty of survival threats and future life directions faced by individuals, who are more likely to develop fast life strategies to perpetuate their survival. Specifically, for violent juvenile offenders, early adversity is likely to cause them to fall into self-identity confusion, be unable to carry out goal establishment, and lose control over their lives [47]. In addition, in this period, violent juvenile offenders still lack an abstract and logical way of thinking about problems, and their dealing with problems carries a certain level of blindness, subjectivity, and emotionality. When facing adverse circumstances, they are more likely than others to develop hedonistic behaviors and tend to develop quick life history strategies to obtain higher gains [48]. This fast life history strategy encourages individuals to adopt an adversarial schema in interacting with society, which is reflected in more risk and aggression [8, 27]. In the long run, this can lead to aggression and even violent crime.

### The moderating role of meaning in life

The results of the moderating effect test found that meaning in life did not moderate the relationship between early adversity and aggression, suggesting that the protective effect of meaning in life is limited and supports the attenuated model.

The test results of the regulatory effect also found that meaning in life moderate the relationship between early adversity and life history strategies. Specifically, the meaning in life is not sufficient to buffer the negative impact of early adversity on individual life history strategies. Its protective effect is weakened at high early adversity levels and stronger at low early adversity levels (strengthening the connection between low early adversity and slow life history strategies). This result supports the attenuated model, which also supports the limited protective effect of meaning in life under high early adversity levels. This research result is consistent with Zheng’s study [38]. A possible explanation for this result is that the meaning in life itself includes values, a sense of purpose, goals, and the ability to integrate the past, present, and future [49], and individuals with a high sense of meaning often have higher future time insights and are more inclined to pursue long-term goals [50, 51]. As the level of early adversity decreases, individuals with a high meaning in life usually have a more positive attitude towards the present and future compared to individuals with a low meaning in life, such as higher life satisfaction and subjective well-being, and a sense of hope [52, 53]. This positive attitude encourages individuals to pursue longer-term goals, have a higher sense of control and value [54], Instead of leaning towards immediate satisfaction, it further guides individuals to adopt more positive coping styles to adapt to life or guide their own actions, resulting in slower life history strategies [55].

### Limitations and future directions

There are also limitations to this study. First, all data were collected through a self-report questionnaire, which can lead to reporting bias. Future studies should combine this with other methods to measure aggression and life history strategies. Second, this study has a cross-sectional research design. The results still do not allow for determining causal relationships or long-term effects among variables. In future studies, longitudinal or experimental designs could be considered to analyze the relationships between variables in more detail. Third, this study discusses only a positive factor (meaning in life) of the personal system in the formation mechanism of violent juvenile offenders’ aggression. Follow-up studies should also focus on the influence of positive aspects in the environment, such as receiving social support. Finally, participants are required to report their experiences of adversity before the age of 12. Considering the age range of 14–25 years, this may leave a long period of time during which other factors may play a role. Future research can also pay closer attention to the nature, duration, chronicity, and severity of adverse experiences.

## Conclusions

This study explored a moderated mediation model that reveals the relationship between early adversity and aggression in violent juvenile offenders, the mediating role of life history strategy, and the moderating role of meaning in life. Possible explanations for the results of this study indicate future research possibilities. Additionally, this study is relevant for those seeking to prevent aggression in adolescents. First, when prison authorities conduct psychological screening, they should anchor the priority population: they should focus on offenders who have negative experiences during childhood. Second, during prison rehabilitation, career planning and interest guidance should be provided to adolescents to help them develop appropriate life history strategies. Finally, attention should be given to the special role of meaning in life among this population.

## Data Availability

The datasets used and analysed during the current study are available from the corresponding author on reasonable request.
